# Different Toll-Like Receptor Expression Patterns in Progression toward Cancer

**DOI:** 10.3389/fimmu.2014.00638

**Published:** 2014-12-15

**Authors:** Lauri Jouhi, Suvi Renkonen, Timo Atula, Antti Mäkitie, Caj Haglund, Jaana Hagström

**Affiliations:** ^1^Department of Otorhinolaryngology, University of Helsinki and Helsinki University Hospital, Helsinki, Finland; ^2^Department of Surgery, University of Helsinki and Helsinki University Hospital, Helsinki, Finland; ^3^Department of Pathology and Oral Pathology, Haartman Institute, Institute of Dentistry and HusLab, University of Helsinki and Helsinki University Hospital, Helsinki, Finland

**Keywords:** innate immunity, carcinoma, pre-malignant lesions, pattern-recognizing receptors, immunohistochemistry

## TLRs in Healthy Tissue

Pattern recognition forms the basis of the innate immune system, allowing it to maintain systemic homeostasis by rejecting harmful molecular structures. Immune cells as well as epithelial cells located near the host–environment boundary, express pattern-recognizing receptors, the activation of which launches cascades leading to immune response and apoptosis (1, 2). Human toll-like receptors (TLRs) 1–10, a family of trans-membrane receptor proteins comprising one type of pattern-recognizing receptors, participate in these immunological processes. In healthy immune cells and epithelial cells, TLR 1, 2, 4, 5, 6, and 10 are usually expressed on the cell surface, whereas TLR 3, 7, 8, and 9 are mainly expressed on the surfaces of endosomes, lysosomes, and endoplasmic reticulum (3). Microbial molecular structures activating pattern-recognizing receptors are called pathogen-associated molecular patterns (PAMPs) (1, 2). Besides exogenous structures, also endogenous molecules, released, for example, from inflamed or damaged tissues, can activate pattern-recognizing receptors and are called danger- or damage-associated molecular patterns (DAMPs) (4, 5). Ligands binding to TLR lead to the activation of several intracellular signaling pathways, activating, for instance, nuclear transcription factors that initiate host defense functions via secretion of co-stimulatory factors and cytokines (6). TLR activation leads also to regulation of gene expression, cell proliferation, differentiation, mitosis, cell-cycle regulation, and apoptosis (7).

## TLRs in Abnormal Conditions

In healthy tissues, TLRs participate in the initiation of inflammatory defense mechanisms, but under abnormal conditions their activation may lead to the process of inflammation becoming chronic. Chronic inflammation is a favorable environment for tumor initiation and progression in multiple organs, including the cervix, stomach, colon, and liver (8–11). In several pre-malignant and malignant conditions, TLRs have been overexpressed (12–15). TLRs can activate immunity affecting proteins like nitric oxide synthase 2 and cyclooxygenase 2, leading to increased malignant potential of cancer cells (12, 16). TLRs can also up-regulate immunosuppressive agents like vascular endothelial growth factor, and transforming growth factor beta within the tumor microenvironment and thus modulate immunity. These modifications can lead to increased angiogenic and metastatic potential of tumors (17). The role of TLRs in cancer is, however, controversial. Activation of TLRs can also lead to tumor inhibition (18–21). The role of TLRs in cancer is therefore ambiguous, as they can on the one hand mediate signaling leading to inhibition of apoptosis and disrupted cell proliferation, and on the other hand, activate immunologic responses against cancer.

Patterns of TLRs expression in terms of frequencies and their subcellular locations are different in cancer microenvironment and in healthy tissue. Here, we discuss the observations of TLR expression patterns in normal, pre-malignant, and carcinoma tissues.

## Transformation of TLR Expression in Cancerous Progression

Under normal circumstances, TLR 2, 4, and 5 are expressed principally on the membrane. During the transformation toward dysplasia, their expression becomes stronger and more cytoplasmic.

The transforming expression pattern has been shown, for example, in the situation of TLR 5 and normal esophageal epithelium transforming toward cancer. In non-dysplastic esophageal epithelium, TLR 5 expression is located exclusively in basolateral plasma membrane and basal cytoplasm (15). During the progression toward dysplasia, the polarity of TLR 5 expression ceases and expression becomes more diffuse. In esophageal adenocarcinoma, the intensity of TLR 5 staining is weaker, when compared to that of columnar epithelial dysplasia, but it follows identical diffuse staining pattern (15). In oral epithelium, TLR 5 expression is already cytoplasmic in healthy tissue but its expression in oral epithelial cancer has been stronger than in healthy epithelium (22).

The phenomenon of stronger and more diffuse TLR expression has appeared also in other TLR subtypes and cancer types. Healthy colon mucosa expresses only minor TLR 4 positivity. In colon dysplasia and adenocarcinoma, TLR 4 expression becomes more evident and is strong and diffuse both in cytoplasm and on membranes (23). TLR 2, 4, and 5 follow the same diffuse cytoplasmic pattern in colon adenocarcinoma, TLR 2 and 4 in gastric columnar epithelial dysplasia, and adenocarcinoma, and TLR 2 and 4 in follicular thyroid carcinoma (24–26).

In colon and gastric columnar epithelial dysplasia, the level of a TLR antagonist, toll-interacting protein (TOLLIP) decreases, whereas TLR expression transforms toward being more diffuse and cytoplasmic. The normal TOLLIP pathway leads to the degradation of excess TLRs. Disruption of the TOLLIP pathway results in excess and altered TLR expression (24, 25).

When DAMPs are bound to plasma membrane or as the cell loses its plasma membrane integrity, and they are released to extracellular matrix, the immune response is initiated (27, 28). DAMPs are able to interact with the immune system to cause tumor suppression, but on the other hand, they can also contribute to cancer progression (29).

DAMPs are confined to the cytosol and nucleus of healthy cells (28). In cancer cells, however, they can be overexpressed, and their intracellular localization can become altered. For example, in cancer, the normally nuclear DAMP-molecule HMGB1 is overexpressed in cytoplasm (30, 31). TLRs 2, 4, and 9 are target receptors for HMGB1 and their activation can launch cascades leading, for instance, to induction of pro-inflammatory cytokines, promotion of angiogenesis, and stimulation of cell migration via downstream intracellular pathways (32). Previously, TLRs that are overexpressed in cytoplasm are suggested to function as a reserve for greater redistribution to the plasma membrane, where they are activated by their extracellular ligands, such as bacteria (24, 25). We could, however, hypothesize that TLRs that are overexpressed and relocated to the cytoplasm in cancer might also be activated in the cytoplasm by their DAMP-ligands, which are equally overexpressed and relocated in to the cytoplasm. This kind of abnormal and uninterrupted intracellular TLR activation could lead to tumor progression in addition to normal inflammatory cascades, although the role of cytoplasmic TLRs needs further study.

## Conflict of Interest Statement

The authors declare that the research was conducted in the absence of any commercial or financial relationships that could be construed as a potential conflict of interest.
